# Statistical Methods for Predicting Malaria Incidences Using Data from Sudan

**DOI:** 10.1155/2017/4205957

**Published:** 2017-03-07

**Authors:** Hamid H. Hussien, Fathy H. Eissa, Khidir E. Awadalla

**Affiliations:** ^1^Department of Mathematics, College of Science & Arts, King Abdulaziz University, P.O. Box 344, Rabigh 21911, Saudi Arabia; ^2^Faculty of Medicine and Health Sciences, University of Kordofan, P.O. Box 160, El-Obeid, Sudan

## Abstract

Malaria is the leading cause of illness and death in Sudan. The entire population is at risk of malaria epidemics with a very high burden on government and population. The usefulness of forecasting methods in predicting the number of future incidences is needed to motivate the development of a system that can predict future incidences. The objective of this paper is to develop applicable and understood time series models and to find out what method can provide better performance to predict future incidences level. We used monthly incidence data collected from five states in Sudan with unstable malaria transmission. We test four methods of the forecast: (1) autoregressive integrated moving average (ARIMA); (2) exponential smoothing; (3) transformation model; and (4) moving average. The result showed that transformation method performed significantly better than the other methods for Gadaref, Gazira, North Kordofan, and Northern, while the moving average model performed significantly better for Khartoum. Future research should combine a number of different and dissimilar methods of time series to improve forecast accuracy with the ultimate aim of developing a simple and useful model for producing reasonably reliable forecasts of the malaria incidence in the study area.

## 1. Introduction

Sudan is one of the most geographically diverse and largest African countries, covering a total area of 1.9 million square kilometers and the population is about 42 million people; most of them live in rural areas. They are considered as a lower-middle income nation, 47% of them living below the poverty line. The gross domestic product is about US $62 billion in 2010 [1]. Malaria is a major public health problem in Sudan. The disease is endemic in the country with varying degree from hypoendemic to holoendemic [2]. Transmission of malaria follows the rainfall season (July to September) in most of the country, except in urban cities and irrigated areas adjacent to the River Nile. The response to malaria has been hindered by a number of factors, for example, conflict, poverty, increased rainfall, the spread of irrigated agriculture within the city limits, poor infrastructure, poor education, the influx of refugees, and insufficient supply of drugs.

Based on WHO estimation in 2011, the number of confirmed malaria cases in the country was 497946 cases [3]. The disease accounted for 37.2% of all maternal deaths at hospital level [4]. It is reported that those affected with malaria were unable to work for 22% of the time during the year [5]. The burden of malaria is very high with the highest rates of morbidity and mortality reported in children [4]. Due to the effectiveness of malaria control program, the burden of malaria has been declined gradually over the last recent years from 1,465,496 total cases in the year 2010 to 945,798 in the year 2012 (Federal Ministry of Health, Malaria National Control Program) [6]. However, Sudan is classified as having insufficient progress in achieving the Millennium Development Goals, where the levels of child and infant mortality are among the highest in the region and the world [7, 8].

Four malaria strata can be identified in Sudan: seasonal transmission (Central Sudan), desert-fringe (Northern part), irrigated (where there is permanent irrigation), and urban malaria (state capital). Accordingly, the risk of malaria transmission varies from 1% to 74% based on climatic condition.

Over the past decades, many new statistical models for predicting the occurrence of malaria epidemics several months in advance have been developed. Comez-Elipe investigates the relationship between environmental factors and disease dynamics and developed a mathematical model to predict malaria incidence in an area of unstable transmission. He provides a useful model for producing reasonably reliable forecasts of the malaria incidence rate [9]. Exponentially weighted moving average models, autoregressive integrated moving average (ARIMA) models with seasonal components, and seasonal multiplicative autoregressive integrated moving average (SARIMA) models were applied on historical malaria morbidity data to examine their ability to predict the number of malaria cases several months in advance. The best model for forecasting and the forecasting error varied strongly between the area of study and the rainfall as a factor was found to improve prediction of ARIMA models in some areas; however, it worsened prediction in other areas [10]. This finding was supported by a study conducted by Briert, in which he found that the addition of rainfall as a factor improved prediction of ARIMA models moderately in some areas but worsened prediction in other areas [10]. Sriwattanapongse and Khanabsakdi used the simplest model for malaria prevalence to provide a malaria prediction model in Northern Thailand. Their model was based on linear regression with the dependent variable defined as the malaria incidence rate in age group effect, period effect with the half year, and cell indexed by district effect. The model provides the best fit to age group, districts, and period [11].

Ordinary Least Square regression method and Generalized Maximum Entropy method were used to examine the factors of malaria incidence in Ghana in 2008. Significant correlation between malaria incidence and climate factors (humidity and temperature) is found. Moreover, the total number of rainy days and humidity is found to be important in predicting malaria incidence [12].

To deal with the data and methodological issues associated with predicting malaria incidence from historical morbidity malaria data, a wide variety of methods have been applied over the recent years [13–15]. In this study, we examine four different approaches (methodologies) of time series analysis to explore whether they can be used to predict malaria incidences with acceptable accuracy from the patterns of historical data alone. Our aim was to find a simple applicable and accurate model in predicting malaria incidences which may be very helpful for the health authority in Sudan to understand the severe and immediate risk of malaria epidemic and to make decisions on effective control actions. We used monthly incidences data collected from epidemic borne states with the unstable transmission of malaria.

## 2. Material and Methods

### 2.1. Data Collection and Study Area

Malaria is considered endemic disease in Sudan. All registers from health centers/units and hospitals throughout the states of the country compiled at the national level to describe malaria situation. In this paper, we are using of data presented by the Ministry of Health between January 2009 and December 2013. The data consist of malaria cases reported in five states: Khartoum, Northern, North Kordofan, Gadaref, and Gazira. These states are known as the most states susceptible to malaria for hundred years back.

In the years 1981, 1988, 1994, 1998, 2003, and 2009 the number of malaria reported cases from Khartoum State was so high, more than the expected number, so they went to be introduced as epidemic years.

The watery nature of Gezira state makes the habitat of the disease since the area represents a good place for mosquitos' proliferation. Irrigated area of Gezira Agricultural Scheme helps in the reproduction of the disease in which malaria is stratified as mesoendemic to hyperendemic with some variation in transmission pattern and occurs year-round with one peak, from August to December with* Plasmodium falciparum* as the predominant species. In 1974-75 severe malaria epidemic affected the Gazira area in the central region. This leads to the establishment of Blue Nile Health Project in 1975, shared between the Sudan government and WHO, World Bank, Kuwait, Japan, and USA. The project addresses malaria control as one of its main issues. And hence malaria was successfully controlled for 10 years and it has been reduced from 25% to less than 1%. Unfortunately, fund stopped and duly, controlling malaria lost the compass and automatically stopped in 1989. The transmission of the disease took an epidemic from the due reduction of local population immunity; the incidence of the disease was again built up to appear in a dramatic epidemic in 1993-94. Also, an outbreak again happened in 2003.

Malaria usually spreads immediately after rain season in Gadaref State, as in 1993 and 1998. Epidemic took place in 1978 in this state following the war in the Ethiopia and due to the mass population movement across the border to the Gadaref State. The epidemic in Northern State is related to heavy floods of the Nile as what happened in the years 1974, 1988, 1989, and 1994. In the western part of Sudan, North Kordofan State resists with a very low health indicator and rates for maternal and infant mortality are high, especially in rural areas [13]. The epidemic had been reported in 1988, 1999, and 2003.

### 2.2. Forecasting Methods

The study was conducted by using E-Views software package to develop ARIMA models. ARIMA model was analyzed with the application of Box-Jenkins approach in which the data was analyzed and used to identify, estimate, and select the best model. First, we check the data whether a series is stationary or not before using it to develop ARIMA models. Four approaches are used to transform a trending series to stationary form. Augmented Dickey-Fuller Test was used to test the null hypothesis (*H*_0_) that the data needs to be differenced to make it stationary versus the alternative hypothesis that the data is stationary and does not need to be differenced [16].

An important practical issue for the implementation of the ADF test is the specification of the lag length. Second, once stationarity is achieved with a determined ARIMA parameter *d* (the number of times the series is differenced to achieve stationarity) we identify the order of the two processes that construct ARIMA model (i.e., AR and MA). Third, to estimate the parameters of the models, we may include as many MA and AR terms as we want in the equation. For example, to estimate a second-order autoregressive and first-order moving average error process, we would use AR(1), AR(2), and MA(1). We need not use the terms consecutively. For example, if we want to fit a fourth-order autoregressive model to take account of seasonal movements, we could use AR(4) by itself. Finally, in order to select an appropriate subclass of models from the general ARIMA (*p*, *d*, *q*), the following approaches of the ARIMA model were used to develop a model to forecast malaria incidence from historical morbidity pattern in five states in Sudan.

#### 2.2.1. Autoregressive Integrated Moving Average (ARIMA)

The autoregressive integrated moving average (ARIMA) models, or Box-Jenkins methodology, are a class of linear models that use historical values of a single variable to forecast its future values; hence they are classified as univariate methods. The model is capable of representing stationary as well as nonstationary time series. However, for adequate ARIMA modeling, a time series should be stationary with respect to mean and variance [17]. For stationary time series, the analysts have to resort to preliminary transformations, such as a log to the original time series, time series differencing, or variance stabilizing to achieve stationarity. Once a stationary series has been obtained a satisfactory model has been obtained and can be used to forecast expected numbers of cases for a given number of future time intervals.

Consider a discrete time series of equally spaced *n* observations in time:

(1) $$\begin{array}{c} Y_{t}=Y_{1},Y_{2},Y_{3},\ldots ,Y_{n-1},Y_{n}. \end{array}$$

An equation of ARIMA model is combining two processes: the autoregressive (AR) process which expresses *Y*_*t*_ as a function of its past values and the moving average (MA) process which expresses *Y*_*t*_ as a function of past values of the error term *e*:

(2) Yt=θ1Yt−1+θ2Yt−2+⋯+θpYt−p−et+∅1et−1+∅2et−2−⋯−∅qet−q,

where *θ*s and *∅*s are the coefficients of the AR and MA processes, respectively, and *p* and *q* are the number of past values of *Y*_*t*_ and the error term used, respectively.

The general notation of ARIMA models is ARIMA (*p*, *d*, *q*), where “*p*” is the order of the autoregressive component, “*d*” is the order of differencing used, and “*q*” is the order of moving average component in the model. Depending on the above definition, the ARIMA models can be classified into the following.

*(a) Autoregressive (AR) Models*. When the value of the current output *Y*_*t*_ depends solely on *p* prior outputs and the current input (random shock) *e*_*t*_, the Box-Jenkins model takes the form of

(3) $$\begin{array}{c} Y_{t}=\emptyset_{1}Y_{t-1}+\emptyset_{2}Y_{t-2}+\cdots +\emptyset_{p}Y_{t-p}+e_{t} \end{array}$$

and is called an autoregressive model of order *p*, denoted by AR(*p*).

*(b) Moving Average (MA) Models*. When the current output *Y*_*t*_ depends solely on the current input and *q* prior inputs, the Box-Jenkins model takes the form of

(4) $$\begin{array}{c} Y_{t}=e_{t}-\theta_{1}e_{t-1}-\theta_{2}e_{t-2}-\cdots -\theta_{q}e_{t-q} \end{array}$$

and is called a moving average model of order *q*, denoted by MA(*q*).

#### 2.2.2. Exponential Smoothing

The simple exponential smoothing model is special cases of ARIMA models. It is suitable for forecasting data with no trend or seasonal pattern, although the mean of the data may be changing slowly over time. It is a weighted average procedure with weights declining exponentially as data become older.

The forecast for next period (period *t* + 1) will be equal to a weighted average of a specified number of the most recent observations:

(5) $$\begin{array}{c} {\hat{Y}}_{t}={\alpha Y}_{t-1}+\left(\;1-\alpha \;\right){\hat{Y}}_{t-1}, \end{array}$$

where *α* is a smoothing coefficient whose value is between 0 and 1.

#### 2.2.3. Transformation Model

In this approach, we use the time series of monthly human malaria incidence data which consists of 60 observations which were transformed to normality via the logarithmic transformation and the relative (log) incidence RI is calculated in order to make data in all area in the same scale.

We define RI for month *t* as

(6) $$\begin{array}{c} Y_{t}=\frac{\left(\ln Z_{t}\right)}{A}, \end{array}$$

where *Z*_*t*_ is the number of cases in month *t* and *A* is the overall mean of the log-transformed series used for forecast.

The following methods were used to forecast RI *n* months in advance, that is, to estimate human malaria incidence in month *t* + *n* (denoted by ${\hat{Y}}_{t+n}$).

#### 2.2.4. Moving Average

In this approach, we use a series of an average of a specified number of the most recent observations in order to smooth out the series by filtering out the “noise” from a random number of malaria cases fluctuations. It is a trend-following or lagging indicator because it is based on past observations.

### 2.3. Model Evaluation

After building our models for each state, the final, as well as the most important, step is to test the accuracy of these models and compare them in order to choose the best predictive models. To select the best model we use (1) Akaike information criterion (AIC) to estimate the quality of each model relative to each of the other models; (2) the Mean Absolute Error (MAE) to measure the average magnitude of the errors in the models; (3) the *R*-square to measure the statistical reliability of the model coefficients; (4) the *t*-statistic to test if a coefficient in the model is zero with the probability of drawing a *t*-statistic of the magnitude of the variable. Probability lower than 0.05 is taken as strong evidence of rejection of the null hypothesis that true coefficient is zero. Taking all these measures into account, the best model among all models specified for the data at hand is the one with the lowest (AIC), lowest (MAE), and highest stationary *R*-square.

## 3. Results

In the analysis, we used five methods to predict malaria cases in five different states in Sudan in order to get the best and simplest method for predicting malaria cases.  Table 1 showed the number of malaria cases reported from the five states during the period 2009–2013. Tables 2–6 showed the resulting models, parameter estimates, and fit statistics for malaria incidence.

Overall, 39383 malaria cases were reported from Khartoum State during 2009–2013, and 108006, 53233, 26567, and 25491 malaria cases were reported from Gezira, North Kordofan, Gadaref, and Northern, respectively. In Khartoum states, the highest number of cases happened in the years 2009 (22321 cases) and 2013 (11190 cases). Also, the results showed that the highest number of malaria cases reported from Gezira, North Kordofan, and Gadaref happened in the years 2009 and 2013, while in the Northern State the highest number of cases was reported in the years 2009 and 2010 (Table 1).

**Figure 1:**
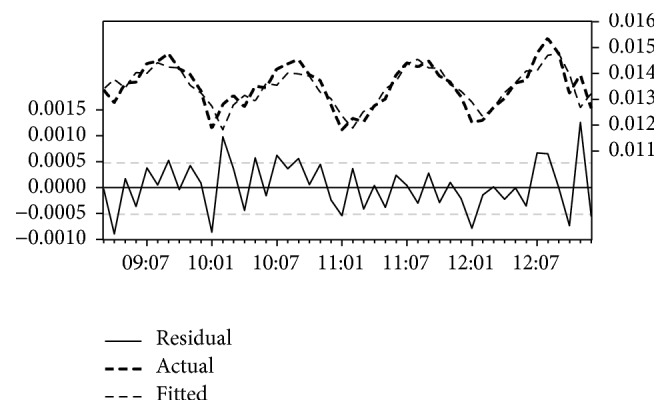
The actual and estimated number of malaria incidences and errors in Gadaref State model.

Table 2 illustrates the result of the analysis of incidence data from Gadaref State. The results show that transformation model with MAE = 0.0008 and AIC = −12.30 tends to provide better forecast performance than the other models for Gadaref State. The estimated coefficients of the model are AR(1) = 1.62; AR(2) = −0.85; MA(1) = −0.96; and constant = 0.013. The model was statistically significant, *P* < 0.001 for all coefficients, and explained 69% of the variance in malaria cases (*R*^2^ = 0.69) in the state. Furthermore, Figure 1 illustrates fitted value, predicted value, and residual value, of the model. The pattern in the lines indicates a clear correlation between the model's predictions and its actual results. Hence this result clearly demonstrates that transformation model is appropriate for this data and best fitted model for malaria forecasting in Gadaref State.

**Figure 2:**
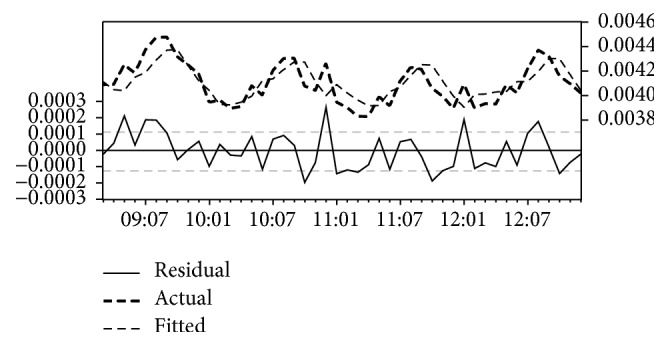
The actual and estimated number of malaria incidences and errors in Gezira State model.

Table 3 illustrates the estimation output of Gezira states' model. The model among all models with the lowest AIC (AIC = −15.12) and lowest MAE (MAE = 9.90*E* − 05) is transformation model.  Figure 2 illustrates fitted value, predicted value, and residual value, of the transformation model. The pattern in the lines indicates a clear correlation between the model's predictions and its actual results indicating that the selected model was the best fit. The estimated coefficients of the model are AR(1) = 1.57; AR(2) = −0.83; MA(1) = −1.04; MA(2) = 0.55; and constant = 0.01. The model was statistically significant, *P* < 0.001 for all coefficients, and explained 55% of the variance in malaria cases (*R*^2^ = 0.55) in the state.  Figure 2 shows a clear correlation between the model's predictions and its actual results.

**Figure 3:**
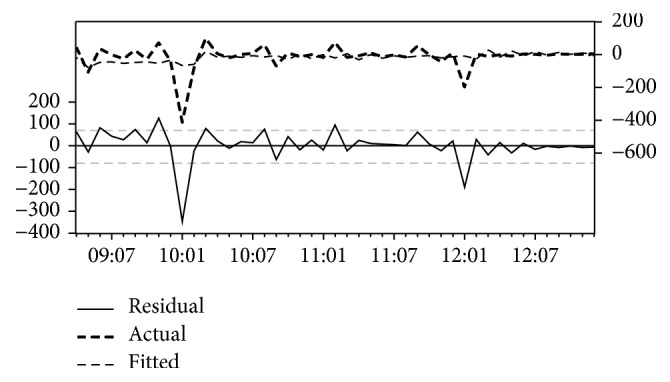
The actual and estimated number of malaria incidences and errors in Khartoum State model.

Table 4 illustrates the estimation output of Khartoum State model. The model with the lowest AIC and lowest MAE is transformation model (AIC = −13.3, MAE = 000). The estimated coefficients of the model are AR(1) = −0.29; AR(2) = 0.17; MA(1) = 0.56; MA(2) = −0.11; and constant = −4.15*E* − 05. However, these entire coefficients are found to be not significant, *P* > 0.001, for all *t*-test statistics and *R*^2^ = 0.07. Moreover, the pattern in the line of actual and predicted values shows a clear instability in two points. Thus the model may not be able to accurately represent the malaria historical data set of Khartoum Statw. The model among all the rest of other models with the lowest AIC and lowest MAE is moving average model (AIC = 11.56, MAE = 53.15) which can be chosen as an alternative model instead of transformation model. AR(1) = −0.28; AR(2) = 0.53; MA(1) = −0.21; MA(2) = −0.77 with (*R*^2^ = 0.12).  Figure 3 illustrates the relationship between the actual and forecasted incidence in Khartoum State.

**Figure 4:**
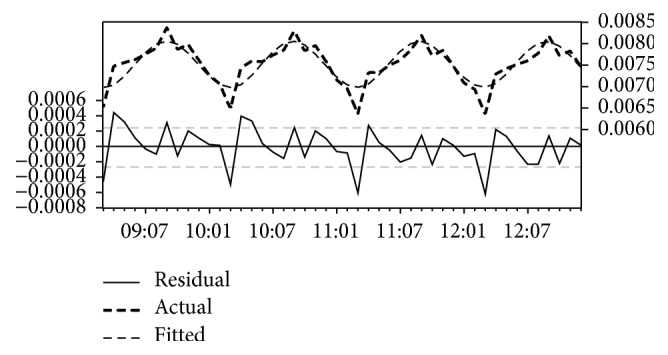
The actual and estimated number of malaria incidence, and errors in North Kordofan State model.

Table 5 portrays the output of the analysis of North Kordofan State time series data. The results, therefore, showed that transformation model has statistically significant coefficients with the lowest AIC and lowest MAE (−13.61 and 0.0003, resp.). The estimated coefficients of the model are AR(1) = 1.73; AR(2) = −1.0; MA(1) = −1.7; MA(2) 0.98; and constant = 0.01. All coefficients were statistically significant, *P* < 0.001, and the high value of the *R*-square thus (0.73) indicates that 73% of the variation in the malaria cases can be explained by the data.  Figure 4 represents the graphical comparison between the actual and forecasted incidence for North Kordofan State time series data series. It is clear that the forecasted series closely resembles the original one. Hence the model could be used for forecasting future malaria incidence in North Kordofan State.

**Figure 5:**
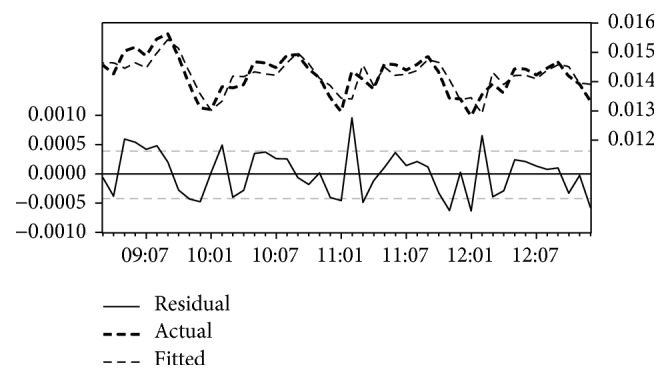
The actual and estimated number of malaria incidences and errors in Northern State model.

Table 6 illustrates the estimation output of Northern State models. The model with the lowest AIC and lowest MAE is transformation model (AIC = −12.67, MAE = 0.0003), with the following coefficients: AR(1) = 0.69; AR(2) = −0.49; MA(1) = 0.25; MA(2) = 0.41; MA(3) = 0.86; and constant = 0.01. The entire coefficients were statistically significant, *P* < 0.001, for all *t*-test statistics. *R*-square is (*R*^2^ = 0.66), indicating that 66% of the variation in the malaria cases can be explained by the data.  Figure 5 illustrate the relationship between the actual and forecasted incidence; the two lines are very close to each other. Hence, transformation model is the one with a good forecasting performance for Northern State.

## 4. Discussion and Conclusion

Models that develop to predict malaria incidence must be simple and understood at least in principle to decision makers in order to be implemented efficiently for future prediction of malaria epidemics [18]. In this paper, we investigate the forecasting capability of ARIMA models. We aim to develop simple models that can predict a future number of malaria incidences from the patterns of historical morbidity data alone by comparing different methods of doing so in terms of the level of accuracy obtained. Four methods of time series have been used in our analysis:

1. Autoregressive integrated moving average (ARIMA)
2. Exponential smoothing
3. Transformation model
4. Moving average.

We used monthly incidence data collected in five states in Sudan with unstable malaria transmission. The analysis was conducted by using E-Views software package. Four models are carried out with stationary series of malaria cases for each of the five states. As it has been understood that for obtaining a reasonable knowledge about the overall forecasting error more than one measure should be used in practice, we have considered two important performance measures for evaluating the accuracy of forecasting models, in particular, AIC and MAE. The best fit model for each state is chosen as an applicable model for the state. Tables 2–6 provide a full comparison of the models. The model evaluation results showed that the transformation model has a smaller MAE for states Gadaref, North Kordofan, Gazira, and Northern while the moving average model has MAE smaller than that for all other models for Khartoum State. This indicates that the transformation model performed significantly better than the other models for states Gadaref, North Kordofan, Gazira, and Northern while the moving average model performed significantly better for Khartoum State. The connection of metrological covariates and malaria epidemics in Khartoum may explain why there is a different model of forecast in this state. Hence, time series study of metrological covariates to forecast malaria incidence in Khartoum is proposed for future research. The suggested models could be easily applicable and understood by decision makers in Sudan. Moreover, our satisfactory understanding about the suggested forecasting models and their successful implementation can be observed from the performance measures and the forecast diagrams for each of the five datasets. However, the analysis of Khartoum incidence data results in nonsignificant coefficients for the transformation model, in spite of the values of AIC and MAE that are lowest in the model compared to those of all other models. In such cases, we can suggest that combined dissimilar methods of time series may improve the forecast performances of malaria incidence in this state. To combine a number of different and dissimilar methods of time series to improve forecast accuracy can provide many scopes for future works.

## Figures and Tables

**Table 1:** The number of malaria cases in the selected states in Sudan during 2009–2013.

Year	Khartoum	Gezira	North Kordofan	Gadaref	Northern
2009	11321	26311	10829	5307	6123
2010	6282	19310	10510	4830	5109
2011	6591	16363	9551	4694	4964
2012	3999	19800	9434	5283	4642
2013	11190	26222	12909	6453	4653

**Table 2:** Models, parameter estimates, and fit statistics for malaria incidence: Gadaref State.

Model	Variable	Coefficient	Std. error	*t*-statistic	Prob.	*R* ^2^	AIC	MAE
ARIMA	*C*	−2.35	22.60	−0.10	0.918	0.10	12.55	92.15
	AR(1)	−0.74	0.16	−4.60	<0.001			
	AR(2)	0.18	0.16	1.14	0.263			
	MA(1)	0.95	0.03	30.17	<0.001			

Exponential smoothing	*C*	421.90	4.02	104.94	<0.001	0.71	11.93	92.43
	AR(1)	1.73	0.07	25.11	<0.001			
	AR(2)	−0.96	0.07	−13.82	<0.001			
	MA(1)	−1.30	0.17	−7.49	<0.001			
	MA(2)	0.34	0.169	2.11	0.041			

Transformation	*C*	0.013	2.76*E* − 05	486.57	<0.001	0.69	−12.30	0.0008
	AR(1)	1.62	0.087	18.73	<0.001			
	AR(2)	−0.85	0.089	−9.62	<0.001			
	MA(1)	−0.96	0.02	−36.79	<0.001			

Moving average	*C*	413.36	27.88	14.83	<0.001	0.31	10.94	44.67
	AR(1)	0.88	0.12	7.39	<0.001			
	MA(1)	−0.56	0.20	−2.82	0.007			

**Table 3:** Models, parameter estimates, and fit statistics for malaria incidence: Gezira State.

Model	Variable	Coefficient	Std. error	*t*-statistic	Prob.	*R* ^2^	AIC	MAE
ARIMA	*C*	1560.10	48.40	32.23	<0.001	0.59	14.73	346.56
	AR(1)	1.59	0.11	14.85	<0.001			
	AR(2)	−0.69	0.10	−6.68	<0.001			
	MA(1)	−0.98	0.04	−22.81	<0.001			

Exponential smoothing	*C*	1554.54	25.91	60.00	<0.001	0.57	14.76	362.60
	AR(1)	1.60	0.11	14.65	<0.001			
	AR(2)	−0.67	0.10	−6.46	<0.001			
	MA(1)	−0.99	0.03	−38.05	<0.001			

Transformation	*C*	0.01	3.42*E* − 05	119.94	<0.001	0.55	−15.12	9.90*E* − 05
	AR(1)	1.57	0.13	12.28	<0.001			
	AR(2)	−0.83	0.12	−6.98	<0.001			
	MA(1)	−1.04	0.17	−6.02	<0.001			
	MA(2)	0.55	0.17	3.27	0.002			

Moving average	*C*	−19.60	14.38	−1.36	0.181	0.32	13.56	153.40
	AR(1)	0.81	0.13	6.17	<0.001			
	AR(2)	−0.75	0.10	−7.38	<0.001			
	MA(1)	−1.44	0.17	−8.43	<0.001			
	MA(2)	1.45	0.14	10.52	<0.001			
	MA(3)	−0.57	0.15	−3.78	<0.001			

**Table 4:** Models, parameter estimates, and fit statistics for malaria incidence: Khartoum State.

Model	Variable	Coefficient	Std. error	*t*-statistic	Prob.	*R* ^2^	AIC	MAE
ARIMA	*C*	404.53	23.61	17.08	<0.001	0.86	12.15	153.65
	AR(1)	1.92	0.16	11.80	<0.001			
	AR(2)	−1.28	0.29	−4.36	<0.001			
	AR(3)	0.31	0.16	1.92	0.063			
	MA(1)	−0.99	0.12	−7.96	<0.001			
	MA(2)	−0.94	0.03	−28.42	<0.001			
	MA(3)	0.93	0.12	7.64	<0.001			

Exponential smoothing	AR(4)	0.49	0.13	3.81	0.001	0.26	12.08	116.09
	MA(4)	−0.94	0.04	−24.03	<0.001			

Transformation	*C*	−4.15*E* − 05	5.70*E* − 05	−0.73	0.471	0.07	−13.30	000
	AR(1)	−0.29	2.42	−0.12	0.906			
	AR(2)	0.17	0.80	0.21	0.839			
	MA(1)	0.56	2.43	0.23	0.820			
	MA(2)	−0.11	1.33	−0.09	0.937			

Moving average	AR(1)	0.28	0.12	2.26	0.028	0.12	11.56	53.15
	AR(2)	0.53	0.21	2.65	0.011			
	MA(1)	−0.21	0.00	−300.98	<0.001			
	MA(2)	−0.77	0.10	−7.49	<0.001			

**Table 5:** Models, parameter estimates, and fit statistics for malaria incidence: North Kordofan State.

Model	Variable	Coefficient	Std. error	*t*-statistic	Prob.	*R* ^2^	AIC	MAE
ARIMA	*C*	841.29	26.04	32.31	<0.001	0.75	13.13	427.14
	AR(1)	1.72	0.01	128.39	<0.001			
	AR(2)	−0.99	0.01	−73.14	<0.001			
	MA(1)	−1.70	0.01	−166.85	<0.001			
	MA(2)	0.98	1.20*E* − 05	81859.26	<0.001			

Exponential smoothing	*C*	62.05	31.98	1.94	0.059	0.45	13.20	211.28
	AR(1)	0.64	0.18	3.47	0.001			
	AR(2)	−0.95	0.23	−4.12	<0.001			
	MA(1)	−0.72	0.384	−1.87	0.068			
	MA(2)	1.54	0.40	3.83	<0.001			

Transformation	*C*	0.01	3.85*E* − 05	195.40	<0.001	0.73	−13.61	0.0003
	AR(1)	1.73	0.01	126.89	<0.001			
	AR(2)	−1.00	0.02	−65.39	<0.001			
	MA(1)	−1.70	0.03	−58.41	<0.001			
	MA(2)	0.98	0.00	334.60	<0.001			

Moving average	*C*	−3.03	0.62	−4.85	<0.001	0.25	9.55	47.95
	AR(1)	0.44	0.14	3.11	0.003			
	MA(1)	−0.97	0.03	−34.56	<0.001			

**Table 6:** Models, parameter estimates, and fit statistics for malaria incidence: Northern State.

Model	Variable	Coefficient	Std. error	*t*-statistic	Prob.	*R* ^2^	AIC	MAE
ARIMA	*C*	404.53	23.69	17.08	<0.001	0.75	11.34	661.05
	AR(1)	1.92	0.16	11.80	<0.001			
	AR(2)	−1.28	0.29	−4.36	<0.001			
	AR(3)	0.31	0.16	1.92	0.063			
	MA(1)	−0.99	0.12	−7.95	<0.001			
	MA(2)	−0.93	0.03	−28.42	<0.001			
	MA(3)	0.93	0.12	7.64	<0.001			

Exponential smoothing	*C*	443.49	18.95	23.40	<0.001	0.87	10.60	50.76
	AR(1)	1.73	0.06	29.87	<0.001			
	AR(2)	−0.99	0.066	−16.42	<0.001			
	MA(1)	−1.09	0.15	−7.35	<0.001			
	MA(2)	−0.69	0.105	−6.67	<0.001			
	MA(3)	1.55	0.17	9.27	<0.001			

Transformation	*C*	0.01	0.00	75.27	<0.001	0.66	−12.67	0.0003
	AR(1)	0.69	0.14	4.76	<0.001			
	AR(2)	−0.49	0.15	−3.27	0.002			
	MA(1)	0.25	0.08	3.075	0.004			
	MA(2)	0.41	0.06	6.96	<0.001			
	MA(3)	0.86	0.08	10.71	<0.001			

Moving average	AR(1)	−0.89	0.22	−4.08	<0.001	0.23	9.85	21.90
	AR(2)	−0.49	0.14	−3.56	0.001			
	MA(1)	0.65	0.23	2.86	0.007			

## References

[1] A-Rahman N. H. A., Jacquet G. A. (2014). The state of emergency care in the Republic of the Sudan. *African Journal of Emergency Medicine*.

[2] http://worldpopulationreview.com/countries/sudan-population/

[3] WHO http://www.emro.who.int/health-topics/malaria/index.html.

[4] Dafallah S. E., El-Agib F. H., Bushra G. O. (2003). Maternal mortality in a teaching hospital in Sudan. *Saudi Medical Journal*.

[5] WHO (2012). *The World Malaria Report 2012*.

[6] WHO (1996). *The World Health Report 1996—Fighting Diseases Fostering Development*.

[7] Bashir A. O., Ibrahim G. H., Bashier I. A., Adam I. (2013). Neonatal mortality in Sudan: analysis of the Sudan household survey, 2010. *BMC Public Health*.

[8] WHO http://www.rollbackmalaria.org/files/files/partnership/wg/wg_monitoring/docs/merg_ConceptualFramework.pdf.

[9] Gomez-Elipe A., Otero A., Van Herp M., Aguirre-Jaime A. (2007). Forecasting malaria incidence based on monthly case reports and environmental factors in Karuzi, Burundi, 1997–2003. *Malaria Journal*.

[10] Briët O. J. T., Vounatsou P., Gunawardena D. M., Galappaththy G. N. L., Amerasinghe P. H. (2008). Models for short term malaria prediction in Sri Lanka. *Malaria Journal*.

[11] Sriwattanapongse W., Khanabsakdi S. (2011). Modelling and forecasting malaria and dengue hemorrhagic fever incidence and prevalence in Northern Thailand. *Journal of Mathematical and System Science*.

[12] Akpalu W., Codjoe S. N. A. (2013). Economic analysis of climate variability impact on malaria prevalence: the case of Ghana. *Sustainability*.

[13] Allard R. (1998). Use of time-series analysis in infectious disease surveillance. *Bulletin of the World Health Organization*.

[14] Helfenstein U. (1991). The use of transfer function models, intervention analysis and related time series methods in epidemiology. *International Journal of Epidemiology*.

[15] Makridakis S. G., Wheelwright S. C., Hyndman R. J. (1998). *Forecasting: Methods and Applications*.

[16] McLeod A. I., Li W. K. (1983). Diagnostic checking ARMA time series models using squared-residual autocorrelations. *Journal of Time Series Analysis*.

[17] WFP (2010). *Emergency Food Security Assessment*.

[18] WHO (2001). *Malaria Early Warning Systems: Concepts, Indicators, and Partners. A Framework for Field Research in Africa*.

